# DNA Methylation Mediates the Association Between Prenatal Maternal Stress and the Broad Autism Phenotype in Human Adolescents: Project Ice Storm

**DOI:** 10.3390/ijms26199468

**Published:** 2025-09-27

**Authors:** Lei Cao-Lei, Guillaume Elgbeili, David P. Laplante, Moshe Szyf, Suzanne King

**Affiliations:** 1 Interdisciplinary School of Health Sciences, Faculty of Health Sciences, University of Ottawa, Ottawa, ON K1H 8L1, Canada; lcao5@uottawa.ca; 2 Douglas Hospital Research Centre, Montreal, QC H4H 1R3, Canada; guillaume.elgbeili@douglas.mcgill.ca; 3 Lady Davis Institute for Medical Research, Jewish General Hospital, Montreal, QC H3T 1E4, Canada; david.laplante@ladydavis.ca; 4 Department of Pharmacology and Therapeutics and Sackler Program for Epigenetics and Developmental Psychobiology, McGill University, Montreal, QC H3A 0G4, Canada; moshe.szyf@mcgill.ca; 5 Department of Psychiatry, McGill University, Montreal, QC H3A 0G4, Canada

**Keywords:** prenatal stress, autism, autistic traits, DNA methylation, epigenetics, adolescence

## Abstract

Prenatal maternal stress (PNMS) predicts risk for autism spectrum disorders (ASD), although the mechanisms are unknown. Because ASD and autistic-like traits have been associated with both prenatal stress and DNA methylation differences, it is important to examine whether epigenetic mechanisms mediate the pathway from PNMS to later autistic-like outcomes. This study aimed to determine the extent to which DNA methylation mediates the association between PNMS from a natural disaster and autistic-like traits in offspring assessed during adolescence. Five months following the 1998 ice storm in Quebec, we recruited women who had been pregnant during the crisis and assessed their PNMS: objective hardship, subjective distress, and cognitive appraisal. At age 13, their children provided blood samples for DNA. At ages 15, 16 and 19, the youth self-reported their own autistic-like traits using the Broad Autism Phenotype Questionnaire. This longitudinal design allowed us to track the developmental pathway from prenatal exposure, through adolescent DNA methylation, to later behavioral outcomes. Analyses included youth with data on PNMS, DNA methylation, and the BAPQ (*n* = 27 at age 15; 22 at age 16; and 13 at age 19). Results showed that mothers’ disaster-related objective hardship and their negative cognitive appraisal of the disaster were associated with DNA methylation at age 13, which then were associated with the severity of their children’s Aloof Personality and Pragmatic Language Deficits, but not Rigid Personality, at ages 15, 16 and 19. Mediation was significant particularly through genes within the PI3K/AKT/mTOR pathway, which has been implicated in various neurodevelopmental disorders, including ASD. Interestingly, while greater PNMS predicted more severe ASD traits, the epigenetics effects were for less severe traits. Although other interpretations are possible, these results could suggest that DNA methylation, assessed in early adolescence, may protect against ASD traits at later ages, particularly when there is a mismatch between the prenatal environment (disaster) and the postnatal environment (absence of disaster). The interpretation of these findings benefits from the longitudinal design and is discussed in the context of fetal programming and the predictive adaptive response.

## 1. Introduction

Autism spectrum disorder (ASD) comprises a set of neurodevelopmental conditions marked by deficits in social interaction and communication, along with restricted and repetitive behaviors [1,2,3]. The broad autism phenotype (BAP) encompasses three primary domains conceptualized along a continuum that are linked to autistic-like traits: aloof personality, pragmatic language impairment, and rigid personality [4,5,6]. These BAP traits are milder yet qualitatively resemble the three core domains defining ASD [7,8,9,10].

Previous research on fetal programming, the developmental origins of health and disease [11,12], and prenatal stress of various kinds, such as maternal depression [13,14,15] and anxiety, or stressful life events [16,17], has consistently demonstrated significant associations with child development [18]. While maternal mood during pregnancy has been associated with increased risk of autism and autistic-like traits in children [19,20].Prenatal maternal stress (PNMS) studies of pregnant women during natural disasters have also shown significant associations with child emotional problems, but with fewer potential confounding factors [21,22,23,24].

The molecular mechanisms underlying the adverse effects of PNMS remain poorly understood [25,26]. One proposed mechanism is the epigenetic modification of gene function. Among these modifications, DNA methylation has been extensively studied, and various maternal experiences are believed to influence epigenetic profiles in the offspring. DNA methylation can alter gene expression without changing the DNA sequence. It represents a plausible biological pathway linking prenatal stress exposures to long-term neurodevelopmental outcomes, including autism-related traits. This epigenetic mechanism may play a role in adapting the genome to stress signals across multiple tissues, thereby accounting for the widespread impact of early life stress on the fetus. Accordingly, epigenetic processes like DNA methylation are strong candidates for explaining how PNMS increases risk for autistic-like traits, while also allowing for individual variability in developmental outcomes.

The PI3K-AKT/mTOR pathway plays a crucial role in regulating cell growth, proliferation, and survival, and its dysregulation has been implicated in various neurodevelopmental disorders, including ASD [27,28,29,30,31]. Recent studies have highlighted the potential for targeting this pathway in the treatment of ASD [32], potentially through pharmacological interventions to modify underlying molecular abnormalities. For example, mTOR inhibitors, such as rapamycin, have shown promise in preclinical models by normalizing synaptic function and improving behavioral outcomes [33]. Additionally, therapies aimed at enhancing PI3K/AKT signaling could potentially restore impaired synaptic plasticity and neuronal connectivity, offering new therapeutic avenues [34].

Although the randomization that is integral to the experimental method used with laboratory animals is unavailable to human researchers studying PNMS, natural experiments can approximate random conditions. Project Ice Storm followed a cohort of children whose mothers were pregnant during one of the worst natural disasters in Canadian history—the 1998 Quebec ice storm [35,36,37]. The strength of this study is that the stressor itself is “independent” of parental psychosocial characteristics and, thus, approximates random assignment to stress conditions [38]. Of interest for studying the development of ASD, we showed, in an earlier analysis, that higher PNMS derived from the ice storm predicted more pronounced autistic-like traits in children at 6½ years old [39]. Recently, Li et al. (2023) reported that PNMS from the ice storm significantly predicted more severe BAP traits in Project Ice Storm youth at age 19, with various aspects of PNMS (objective hardship, subjective distress, and cognitive appraisal) accounting for up to 21.4% of the variance in BAP traits [40].

Several epigenetic findings from Project Ice Storm have reported that different components of PNMS triggered genome-wide DNA methylation changes in the children (see review [41]), suggesting that DNA methylation is a potential mechanism through which PNMS affects child development. However, the extent to which such methylation changes are linked specifically to later autistic-like traits has not been tested. This knowledge gap provides a strong rationale for examining whether DNA methylation mediates the association between PNMS and BAP traits. Additionally, the long-term follow-up of children from studies like Project Ice Storm is needed to determine the lasting effects of these epigenetic changes on physical and mental health outcomes.

**Figure 1:**
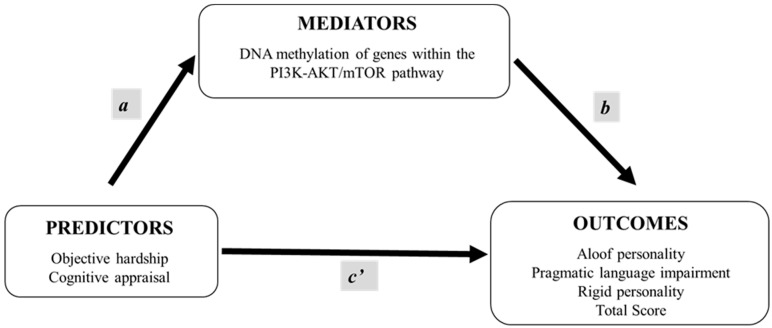
Mediation model. Coefficient *a* represents the effect of the predictor variables (prenatal maternal stress) on the mediator variables (DNA methylation at age 13), while coefficient *b* signifies the effect of the mediator variables on outcome variables (BAPQ scores at ages 15, 16, and 19 years), controlling for the predictor variables. Coefficient *c’* is the direct effect of the predictor variables on the outcome variables, controlling for the mediator variables.

The objective of this study was to determine the mediating role of DNA methylation during early adolescence in the relationship between ice storm-related PNMS and BAP traits in offspring during mid-to-late adolescence. By focusing on epigenetic modifications, specifically DNA methylation of genes within the PI3K-AKT/mTOR pathway, we aimed to elucidate the molecular mechanisms through which PNMS influences autism-like traits. We hypothesized that two components of PNMS (objective hardship and cognitive appraisal), which are both associated with alterations in DNA methylation in this cohort, would mediate the expression of BAP traits in children at 15, 16, and 19 years of age (Figure 1). Specifically, we expected that different aspects of PNMS would have distinct impacts on DNA methylation patterns and consequently on the severity of BAP traits, including aloof personality, pragmatic language impairment, rigid personality and the total score, across all three ages. By addressing these objectives, this study seeks to advance our understanding of the epigenetic mechanisms underlying the impact of PNMS on neurodevelopment.

## 2. Results

### 2.1. Descriptive Statistics

Of the 34 mothers whose children participated in the epigenetic study, none rated the consequences of the storm as “Very negative”, 12 (35.3%) rated them as “Negative”, 4 (11.8%) as “Neutral”, 17 (50.0%) as “Positive”, and 1 (2.9%) as “Very positive”. Since our focus was on the effect of negative cognitive appraisal of the ice storm on child outcomes, we combined the “Neutral” and “Positive” ratings into a single “Positive” group and reversed the recoded scores as follows: (1). Very positive, (2). Neutral/Positive, (3). Negative, (4). Very negative.

The study sample sizes included 27 individuals who had both DNA methylation and BAP data at age 15, 22 individuals at age 16, and 13 individuals at age 19. The descriptive statistics for objective hardship, BAPQ are shown in Table 1. None of the PNMS variables (Objective Hardship, Subjective Distress, Cognitive Appraisal) differed significantly between subjects who were retained until age 19 and those who were not retained (*p*-values range from 0.236 to 0.801).

### 2.2. Correlation Analyses

The correlations among the PNMS scores and the BAPQ subscales at ages 15, 16, and 19 are presented in Table 2. In general, there were strong associations among the BAPQ subscales within and across the three ages. Although failing to reach statistical significance, Objective Hardship was moderately associated with rigid personality at all three ages (r > 0.30 n.s.), and with aloof personality at age 16 (r = 0.396, n.s.). Cognitive Appraisal was only associated with rigid personality at age 19 (r = 0.426, n.s.).

### 2.3. Mediation Analyses

Table 3 presents a summary of the mediation results at each of the three ages for all mediations tested between objective hardship or cognitive appraisal and the three subscales of the BAPQ and the BAPQ total score. The table presents the number of CpGs, and the number of genes they correspond to that were associated with the PI3K/AKT/mTOR pathway for either objective hardship or cognitive appraisal and were tested at each age. This information is followed by the number of those CpGs (and the number of genes) whose DNA methylation levels were found to significantly mediate the effect of PNMS on BAPQ scores. The table then presents the minimum and maximum values of the significant mediation effects (indirect effect a ∗ b) as well as their locations, and the mean value of the significant mediation effects and their locations. Finally, the table presents the minimum and maximum effect sizes for significant results, represented as R^2,^ which reflects the percent of variance in the BAPQ score that is explained by both the predictor (path c’) and mediator (path b). The complete results are presented in the Supplemental Tables S2 and S3.

#### 2.3.1. Methylation Levels of CpGs Mediate the Association Between Maternal Objective Hardship and Components of BAP (Table 3)

To summarize the results of the mediation analyses for Objective Hardship, the negative mediation effects indicate that greater maternal objective hardship from the ice storm influences the child’s DNA methylation in ways that are associated with lower scores on BAP traits at all three ages.

The results consistently highlight the role of specific CpGs associated with the PI3K/AKT/mTOR pathway in mediating the association between maternal objective hardship and aloof personality, pragmatic language impairment, and total BAP scores, with varying levels of mediation across different ages. Significant CpGs such as those in NFKBIA, PIK3CD, and RPTOR show consistent involvement. As for rigid personality, there was only a single CpG with a significant mediation effect, which was also in the negative direction.

#### 2.3.2. Methylation Levels of CpGs Mediate the Association Between Maternal Cognitive Appraisal and Components of BAP (Table 3)

Again, the negative mediation effects indicate that more negative maternal appraisal of the ice storm influences the child’s DNA methylation in ways that are associated with lower scores on BAP traits. The methylation levels of multiple CpGs associated with the PI3K/AKT/mTOR pathway significantly mediated the association between cognitive appraisal and aloof personality, pragmatic language impairment, and total BAP scores; only one CpG mediated the association between maternal cognitive appraisal and rigid personality, and that was at age 16. In addition, the only positive mediation effect in these analyses was on pragmatic language impairment: more negative cognitive appraisal predicted more severe impairment as mediated by one CpG in BCL2L1. The percent of variance explained ranged from 10% to 34%, with consistent patterns across various ages. Significant CpGs include those in PIK3R2, PRKCH, and RPTOR, while many other CpGs exhibited no significant mediation effects.

### 2.4. Summary

Taken together, the findings indicate that DNA methylation serves as a significant mediator linking both objective hardship and cognitive appraisal to various components of the broad autism phenotype (BAP) at different ages, with the exception of rigid personality. Maternal objective hardship and cognitive appraisal are both associated with the DNA methylation of genes involved in autism-related pathways, either with decreasing DNA methylation levels of certain genes (a < 0) or with increasing DNA methylation levels of certain genes (a > 0) (Supplementary Tables S2 and S3). The mediation effects are negative, except for one, indicating that the DNA methylation effects are associated with a reduced expression of BAPQ traits. The variance in BAP traits explained by the significant negative mediations ranges from approximately 13% to 41% across different ages and traits; the single result showing a positive mediation effect of cognitive appraisal on pragmatic language impairment explained 10% of the variance in the BAPQ score.

## 3. Discussion

Given that the mechanisms behind the association between PNMS and autism are largely unknown, we aimed to determine the extent to which changes in DNA methylation may be one such mechanism. Our findings demonstrate a significant mediating role of DNA methylation in the relationship between PNMS, such as objective hardship and cognitive appraisal, and various components of the BAP across adolescence. Interestingly, however, rather than explaining how PNMS increases autistic-like traits in offspring, our results demonstrate a mechanism by which epigenetic processes served to dampen the PNMS effects.

The results indicate that maternal objective hardship and cognitive appraisal are associated with DNA methylation levels, which are associated with BAP traits. The negative mediation effects observed for aloof personality, pragmatic language impairment, and total BAP scores across ages 15, 16, and 19 suggest that greater maternal objective hardship, and more negative cognitive appraisal, are associated with either increased or decreased DNA methylation of specific genes within the PI3K/AKT/mTOR pathway in ways that then appear to weaken the effect of PNMS on neurodevelopmental deficits related to autism. This suggests that the methylation of these genes related to the PI3K/AKT/mTOR pathway may play a protective role against early environmental adversity, influencing neurodevelopmental outcomes in adolescence and highlighting the adaptive role of epigenetics in development [42]. This result echoes other Project Ice Storm findings showing that methylation of genes in diabetes mellitus pathways weakened the significant associations between maternal prenatal objective hardship and body mass index (BMI) and central adiposity during early adolescence [43]. Interestingly, a number of genes overlap between the diabetes mellitus and PI3K/AKT/mTOR pathways studied here. CpG sites on the NFKBIA and PIK3CD genes significantly negatively mediated between maternal prenatal objective hardship and BMI [43], as well as between objective hardship and BAP traits. In addition, emerging evidence [43,44,45,46,47] suggests that epigenetic modifications may also serve a protective role, helping to buffer individuals against the long-term consequences of early-life adversity and facilitating more adaptive developmental trajectories. For instance, we reported that increased DNAm in the glucocorticoid receptor gene (NR3C1) buffers the effects of maternal anxiety on child behavioral outcomes in a Dutch cohort [44]. Complementing our findings, research from the ALSPAC cohort demonstrated that epigenome-wide DNAm patterns mediate the link between childhood adversity and depressive symptoms in adolescence, identifying CpGs with apparent protective effects [45]. The same group also showed that adversity-related DNAm differences can exert both risk-enhancing and protective effects, supporting a dual role of methylation in shaping developmental trajectories [46]. Importantly, these patterns are not limited to human studies: evidence from experimental animal models has demonstrated that DNAm can protect against cisplatin-induced kidney injury via regulation of genes such as interferon regulatory factor 8 [47], thereby strengthening the biological plausibility of protective epigenetic mechanisms across species and organ systems. This type of mechanistic evidence suggests that, at least in certain contexts, DNAm may act as an active mediator of biological adaptation to environmental exposures, regulating both risk and resilient phenotypes.

While our study identified numerous negative mediation effects and only a single positive mediation effect, this does not preclude the existence of additional positive mediation effects that were not captured in our analyses. Many CpG sites were not included, and our investigation was limited to blood tissue, without examining (inaccessible) brain tissue, where such effects may be more pronounced [48].

Together, our finding indicates that not all epigenetic changes resulting from maternal hardship are detrimental and that some may confer resilience against neurodevelopmental impairments. Although the relationship is complex and context-dependent, some studies indicate that epigenetic mechanisms can promote resilience, especially when influenced by positive environmental factors, early interventions, or protective gene-environment interactions. For instance, animal studies have shown that early-life maternal care can lead to beneficial epigenetic modifications in the brain regions associated with stress responses (e.g., the *Nr3c1* gene, which regulates glucocorticoid receptors) [49,50,51,52]. These changes can promote resilience and reduce the risk of neurodevelopmental and behavioral issues later in life. The work by Meaney and colleagues demonstrated that higher levels of maternal care in rodents resulted in epigenetic modifications that enhanced stress resilience in offspring, highlighting the role of environmental influences on epigenetic regulation [53,54,55]. Unfortunately, Project Ice Storm does not include measures of maternal care; at any rate, we assume that the random distribution of ice storm-related hardship in the population would also mean that the quality of maternal care would be randomly distributed in our sample.

The negative mediation of DNA methylation seen in our BMI analyses [43] and in the BAP analyses presented here may be the result of more macro-level environmental changes rather than individual levels of maternal care. Epigenetic mechanisms, such as DNA methylation, play a crucial role in shaping genome function in response to these environmental cues. The underlying purpose of this adaptation is to optimize an organism’s fitness and enhance its survival prospects in changing environments. The theory of Predictive Adaptive Response (PAR) [42,56,57] suggests that, during pregnancy, the placenta receives signals from the mother about the nature of the outside world into which the fetus will emerge, and that fetal development is then adjusted to better meet outside environmental threats. Given a mismatch [58] between the environmental conditions during gestation (e.g., a major natural disaster) and the environmental conditions during childhood and later (e.g., no natural disaster), perhaps the subsequent non-threatening environmental signals received by the child resulted in a reversal of the original, PAR adaptations. As such, our data provides suggestive evidence of epigenomic programming of adaptation to the non-threatening postnatal environment. To test this theory, future studies would need repeated epigenetic analyses from birth through adolescence [25].

Interestingly, the only observed positive mediation effect is for cognitive appraisal by way of one CpG site in BCL2L1 (cg08257293) at age 15 in relation to pragmatic language impairment. This is the only result that is in the direction of explaining how PNMS might exacerbate neurodevelopmental deficits related to autism. We note that 3 other CpG sites on the same BCL2L1 gene also have significant mediation effects between cognitive appraisal and pragmatic language deficits, but those effects are in the negative, protective direction. In addition, one of those 3 CpGs (cg13989999) was also a significant mediator of cognitive appraisal on rigid personality, and of objective hardship effects on pragmatic language, as well as on aloof and total BAPQ. Thus, it is difficult to conclude that the gene, in and of itself, has protective value. Therefore, further research is needed to explore the conditions under which maternal hardship and negative cognitive appraisal can lead to beneficial epigenetic modifications.

It is noteworthy that, despite multiple significant mediation effects for aloof personality and pragmatic language impairments, only 2 significant mediation effects were found for rigid personality: one from objective hardship at age 15 (in PRR5L) and one from cognitive appraisal at age 16 (in FNBP1). As seen in Table 2, the strongest correlation between objective hardship and a BAPQ score was with rigid personality at all three ages; this suggests that there may be some other mechanism by which PNMS influences rigid personality in adolescence. Project Ice Storm researchers have been studying associations between PNMS and brain functional connectivity [59]. Rigid personality appears to be a function of both objective hardship and cognitive appraisal as mediated by increased functional connectivity from the right central amygdala to the left inferior lateral occipital cortex [60]. The rigid personality subscale has some of the lowest correlations with the other two subscales, which might suggest the possibility of this dimension having different underlying properties and etiology than the other two. However, it is important to note that the absence of epigenetic mediating effects on the Rigid Personality subscale in blood does not necessarily imply that such effects are nonexistent. First, alternative pathways beyond the PI3K/AKT/mTOR pathway may be involved. Second, other CpG sites not interrogated by the 450K arrays could contribute to these effects. Third, this study examined a single cell source (blood), rather than the brain, where epigenetic effects are most likely to manifest.

Potential limitations of the present study include the relatively small sample size, due to attrition, particularly the diminishing sample size by age 19, which could influence the reliability of our findings. Therefore, we used bootstrapping to boost reliability for mediation analyses. Ideally, however, these findings should be validated in a larger sample. We could not, however, justify testing the moderation of the effects by sex, which would have required considerably more statistical power. As such, our study cannot address the causes of sex differences in the risk for autism. The lack of RNA samples due to the low amount of blood collected hindered our ability to determine the expression levels of PI3K-AKT/mTOR-related genes. While our findings emphasize the potential role of DNA methylation in PI3K/AKT/mTOR-related genes, other biological systems, such as HPA axis function [61] and immune signaling [62] may also contribute to neurodevelopmental outcomes and warrant investigation in future pathway-level analyses. It is important to note that DNA methylation was assessed in peripheral T-cells rather than in brain tissue, where autism-related neurodevelopmental processes primarily occur. Although previous studies [63,64,65] have shown some cross-tissue concordance in methylation patterns, the extent to which peripheral methylation accurately reflects brain-specific epigenetic modifications remains uncertain. Therefore, caution is warranted when interpreting these findings in the context of brain development. Furthermore, the absence of epigenetic data at earlier developmental stages constrains our ability to understand the temporal dynamics of epigenetic modifications and their long-term effects. Future studies with larger longitudinal cohorts are necessary to validate our findings and provide a more nuanced understanding of the role of epigenetics in ASD.

This study possesses several notable strengths that enhance its contribution to understanding the role of epigenetics in autism spectrum disorder (ASD). Firstly, we employed repeated assessments of BAPQ at three ages during mid-to late adolescence. Effects that are replicated across ages are most likely to be important, indicating a consistent pattern that may reflect stable traits or enduring influences over time. As a longitudinal study, it allows for the examination of changes and developments at multiple time points—ages 15, 16, and 19—providing a comprehensive view of the progression and stability of epigenetic modifications and their association with the broad autism phenotype (BAP) over time. Secondly, the integration of both epigenetic and behavioral data enables a robust analysis of the relationships between DNA methylation patterns and BAP traits. The study is among the few that incorporate various aspects of PNMS, including objective hardship as a continuous variable, and cognitive appraisal—factors often overlooked in fetal programming models which typically emphasize maternal mood and stress hormones influencing fetal development through the placenta. Additionally, the inclusion of multiple subscales from the BAPQ (aloof personality, pragmatic language impairment, and rigid personality) allows for a detailed examination of specific domains within the broader autism spectrum as self-reported by the participants. Although a case could be made for the value of measuring the observations of external behaviors from significant others, such as parents, we chose to focus on the internal experience of the young person; an improvement would be to somehow include both self-report and parent-report measures. Lastly, focusing on PI3K/AKT/mTOR pathway-related genes provides valuable insights into the potential molecular mechanisms by which prenatal maternal stress affects neurodevelopment, contributing to the growing body of literature on the epigenetic basis of ASD.

Future research should aim to further explore the temporal dynamics of these epigenetic changes and their long-term effects on neurodevelopmental outcomes through additional longitudinal studies. Investigating the interactions between genetic predispositions and epigenetic modifications could also yield deeper insights into the mechanisms driving autism-related traits. Moreover, exploring potential interventions, such as stress reduction programs for expectant mothers, may prove valuable in mitigating the impact of maternal stress on children’s neurodevelopment by addressing epigenetic modifications.

## 4. Materials and Methods

### 4.1. Participants

Four months after the ice storm, we contacted physicians who deliver babies in the Montérégie region on the south shore of Montreal. They identified a total of 1440 women who were 18 years or older and pregnant on 9 January 1998 (the peak of the ice storm), or who had become pregnant within 3 months of that date. We delivered the requisite number of stamped questionnaire packets to each physician’s office, where they were addressed to each patient. On 1 June 1998, the offices mailed the packets. The 178 women who returned the postal questionnaires to us and agreed to further follow-up were all non-Hispanic White and French-Canadian, consistent with the sociodemographic of the region. Participating families were significantly better educated (33% had a university degree or higher) and had higher income than the average of the region, where 21% of women aged 20–44 had degrees. The women and their children were assessed approximately every two years for nearly twenty years; by age 19, however, there was considerable loss to follow-up.

### 4.2. Questionnaires

All phases of this study were approved by the Research Ethics Board of the Douglas Hospital Research Center in Montreal, Canada. We obtained written informed consent from parents and written assent or consent from adolescents at all assessments.

Objective Hardship was estimated using the mothers’ responses to questionnaire items tapping into categories of exposure used in other disaster studies: Threat, Loss, Scope, and Change. Items asked objective questions such as the dates of power loss and return (scope), if there was damage to the home (loss), if the woman was injured (threat), and the number of times they changed lodging (change). Points were allotted for each response on each item such that each of the four scales was scored with a maximum of 8 points. The sum of the subscales yielded a total Objective Hardship score called Storm32. A list of Storm32 items and their scoring is described elsewhere. All the subscales, except Threat, had excellent test–retest reliability 6 years after the ice storm (intraclass correlations: scope: ƥ = 0.80; change: ƥ = 0.82; and loss: ƥ = 0.69)); Threat had moderate test–retest reliability (ƥ = 0.42). We previously demonstrated that Storm32 was significantly correlated with the DNA methylation in T-cells at 1675 CpG sites affiliated with 957 genes when the youth were 13 years of age [66].

Subjective Distress: We operationalized the mothers’ subjective distress from the disaster as the severity of their post-traumatic stress disorder (PTSD) symptoms related to the ice storm, assessed by the 22-item Impact of Event Scale—Revised (IES-R) [67]. Results from a previous analysis showed, however, that there were no significant correlations between IES-R scores and the children’s DNA methylation [66]. Thus, we have not included the IES-R in the current study.

Cognitive Appraisal: To assess cognitive appraisal, women responded to the following item: “Overall, what were the consequences of the ice storm on you and your family?” Response options were on a five-point scale of “Very negative” (1), “Negative” (2), “Neutral” (3), “Positive” (4), and “Very positive” (5). We demonstrated previously that scores on this item were significantly correlated with the DNA methylation of 2872 CpG sites in Project Ice Storm youth at age 13 years [68].

Autistic-like Traits: The 36-item Broad Autism Phenotype Questionnaire (BAPQ) [5] is a self-report tool developed to assess BAP traits in clinical and non-clinical youth and has been validated against direct clinical assessments of BAP. It has three subscales of 12 questions each, targeting aloof personality, pragmatic language impairment, and rigid personality. Further details have been reported elsewhere [40]. The BAPQ was administered at ages 15, 16, and 19 years.

### 4.3. Blood Collection and DNA Extraction

In 2011, a subgroup of 34 children (19 Boys and 15 girls) from Project Ice Storm agreed to participate in a blood draw that included an epigenetic study. DNA was extracted from T cells and analyzed using the 450K human methylation array. T cells were isolated from peripheral blood mononuclear cells (PBMCs) through immunomagnetic separation with Dynabeads CD3 (Dynal, Invitrogen). DNA was then extracted from these T cells using the Wizard Genomic DNA Purification kit (Promega), following the manufacturer’s guidelines.

At age 15, 27 children had complete data on PNMS, DNA methylation, and the BAPQ, with 22 children at age 16 and 13 children at age 19. When accounting for overlap across assessments, the combined cohorts comprised 27 unique individuals at ages 16 and 19, 29 unique individuals at ages 15 and 16, and 28 unique individuals at ages 15 and 19.

### 4.4. Infinium Human Methylation 450K BeadChip Array and Data Analysis

Infinium HumanMethylation450K BeadChip, an array containing 485,577 probes covering 99% RefSeq genes and 96% of CpG islands, was used to determine DNA methylation levels in T cells. Probes on chromosomes X and Y were excluded. To avoid artifacts due to hybridization bias, probes with minor allele frequency (MAF) ≥ 5% in the HapMap CEU population were removed. Furthermore, CpGs with an inter-quartile range (IQR) less than 0.10 (i.e., 10% methylation difference) were not analyzed. The remaining 10,553 probes were tested for association with objective hardship and cognitive appraisal. The Benjamini–Hochberg algorithm was used to correct for multiple testing by computing the false discovery rate (FDR), which was set at <0.2. Illumina 450K Methylation BeadChip analyses were completed using standard procedures and described previously [66].

### 4.5. Selection of CpGs Within Genes of the PI3K-AKT/mTOR Pathway

In order to determine the extent to which gene methylation mediates the association between objective hardship or cognitive appraisal and BAP, we tested genes that we have previously shown to have their methylation signatures from isolated T cells associated with objective hardship (24 genes) and cognitive appraisal (19 genes) in this sample [66]. We then matched the genes whose methylation had been significantly correlated with objective hardship and/or cognitive appraisal to the PI3K AKT mTOR pathways as classified by IPA software. In total, objective hardship was associated with the methylation levels of 27 CpGs from 18 genes in the PI3K AKT mTOR pathways, and cognitive appraisal was associated with the methylation levels of 61 CpGs from 41 genes. There were 22 CpGs that overlapped in the pathways associated with both objective hardship and cognitive appraisal. As such, the 66 unique CpGs were selected and used for further analysis (Table S1).

### 4.6. Statistical Analysis

All analyses were completed with SPSS 20.0 (SPSS, Chicago, IL, USA).

Pearson product-moment correlations were conducted between the outcome measures and all predictors. Mediation analyses were conducted using state-of-the-art bootstrapping. Mediation models hypothesize a causal chain in which the independent variable affects the mediator variable, which in turn affects the dependent variable. The current mediation analyses included the components of BAP at each of the three ages as the dependent variable, and either objective hardship or cognitive appraisal as the independent variable, and DNA methylation levels of CpGs at age 13 as mediators. We used bootstrap methods, one of the routinely used approaches, to determine the significance of mediation effects in the mediation analyses [69]. Bootstrapping is a powerful approach because it takes into account that the sampling distribution of the mediated effect is skewed away from 0, allowing for more accurate estimation of confidence intervals and significance levels [70], and this approach can be applied for small-to-moderate samples (i.e., sample sizes ranging from 20 to 80 cases) [71]. 95% bias-corrected bootstrap confidence intervals were computed, as explained by Hayes [72]. The PROCESS procedure for SPSS [72] was used to conduct the analyses. Each bootstrap resampled the initial sample 10,000 times. A mediating effect was considered significant if 0 was not included in the bootstrap confidence interval.

## 5. Conclusions

In summary, our study demonstrates that maternal objective hardship and cognitive appraisal are significantly associated with the development of autism-related traits at ages 15, 16, and 19, through DNA methylation modifications measured at age 13. While the initial aim was to test the hypothesis that DNA methylation of selected ASD pathway-related genes would amplify the negative effects of disaster-related PNMS on autistic-like traits, our findings instead suggest the opposite effect of methylation in genes associated with the PI3K/AKT/mTOR pathway, perhaps in response to the non-threatening post-natal environment. These results underscore the critical role of epigenetic mechanisms in shaping the developmental trajectory of BAP components and highlight the importance of addressing multiple facets of maternal disaster-related stress (e.g., objective hardship and cognitive appraisal) to improve neurodevelopmental outcomes. Further research, specifically longitudinal studies that track the methylation of PI3K/AKT/mTOR pathway genes from infancy following a significant prenatal stressor, is needed to deepen the understanding of this unexpected finding.

## Figures and Tables

**Table 1:** Descriptive Statistics.

	Age 15 Years (*n* = 27)				Age 16 Years (*n* = 22)				Age 19 Years (*n* = 13)			
	Mean (or *n*)	SD	Min.	Max.	Mean (or *n*)	SD	Min.	Max.	Mean (or *n*)	SD	Min.	Max.
Sex of child												
Male	15				13				4			
Female	12				9				9			
Objective Hardship	10.9615	3.96465	5.00	21.00	11.2857	4.38341	5.00	21.00	10.1538	3.02341	6.00	15.00
Cognitive Appraisal												
Very Positive	1				1				0			
Neutral/Positive	18				14				8			
Negative	8				7				5			
Very Negative	0				0				0			
BAPQ_Aloof	2.27333	0.600103	1.170	3.180	2.63273	0.902395	1.080	4.750	2.63538	0.933601	1.330	4.420
BAPQ_PragLan	2.37926	0.451407	1.330	3.170	2.42364	0.553186	1.080	3.580	2.15385	0.607447	1.000	2.750
BAPQ_Rigid	2.56741	0.564314	1.000	3.420	2.88591	0.712558	1.750	4.670	2.85846	0.687300	1.920	3.830
BAPQ_Total	2.40667	0.430724	1.470	3.030	2.64773	0.603221	1.610	3.670	2.55000	0.652712	1.530	3.310

Note. PragLan = Pragmatic language impairment.

**Table 2:** Correlations among the prenatal maternal stress variables and the BAPQ subscales at ages 15, 16, and 19.

	1	2	3	4	5	6	7	8	9	10	11	12	13	14
1. Objective Hardship	1													
2. Cognitive Appraisal	0.276	1												
3. K15_BAPQ_Aloof	0.186	0.112	1											
4. K15_BAPQ_PragLan	0.083	−0.069	0.485 *	1										
5. K15_BAPQ_Rigid	0.312	0.140	0.481 *	0.407 *	1									
6. K15_BAPQ_ToT	0.249	0.088	0.842 **	0.751 **	0.800 **	1								
7. K16_BAPQ_Aloof	0.028	−0.191	0.534 *	0.324	0.347	0.548 *	1							
8. K16_BAPQ_PragLan	0.083	−0.174	0.517 *	0.774 **	0.378	0.719 **	0.435 *	1						
9. K16_BAPQ_Rigid	0.394	−0.021	0.410	0.394	0.688 **	0.667 **	0.601 **	0.555 **	1					
10. K16_BAPQ_ToT	0.204	−0.156	0.589 **	0.555 *	0.541 *	0.750 **	0.868 **	0.741 **	0.864 **	1				
11. K19_BAPQ_Aloof	0.370	0.015	0.708 **	0.225	0.471	0.596 *	0.538	0.364	0.626	0.582	1			
12. K19_BAPQ_PragLan	0.051	−0.116	0.659 *	0.754 **	0.620 *	0.827 **	0.518	0.849 **	0.474	0.677	0.650 *	1		
13. K19_BAPQ_Rigid	0.396	0.426	0.202	−0.072	0.644 *	0.350	0.218	−0.044	0.548	0.268	0.709 **	0.555 *	1	
14. K19_BAPQ_ToT	0.329	0.119	0.623 *	0.308	0.659 *	0.672 *	0.501	0.405	0.657	0.586	0.929 **	0.817 **	0.861 **	1

Note. PragLan = Pragmatic language impairment; ToT = Total score. * Correlation is significant at the 0.05 level (2-tailed). ** Correlation is significant at the 0.01 level (2-tailed).

**Table 3:** Summary of significant indirect (mediation) effects from PNMS to BAP dimensions via DNA methylation in genes associated with the PI3K/AKT/mTOR pathway.

					Significant Mediation Effects			Significant Effect Sizes		
			CpGs (Genes)		Range of Significant Mediating Effects			Range of Significant Effect Sizes (R^2^)		
PNMS	BAPQ	Age	Total Tested	Significant Mediating	Minimum (Location)	Maximum (Location)	Mean Mediation	Minimum (Location)	Maximum (Location)	Mean R^2^
Objective Hardship	Aloof	15	27 (18)	20 (15)	−0.0200 (cg08792630 in *FOXO3*)	−0.0361 (cg00689225 in *NFKBIA*)	−0.0284	0.1791 (cg20171453 in *RHOH*)	0.3119 (cg00689225 in *NFKBIA*)	0.2260
		16		18 (13)	−0.0262 (cg17904575 in *PPP2R5C*)	−0.0547 (cg00689225 in *NFKBIA)*	−0.0343	0.0941 (cg01320698 in *PIK3CD*)	0.3823 (cg00689225 in *NFKBIA*)	0.1967
		19		7 (7)	−0.0945 (cg00689225 located in *NFKBIA*)	−0.1161 (cg26360197 located in *RPTOR*)	−0.1028	0.3378 (cg00689225 located in *NFKBIA*)	0.4512 (cg11833768 located in *PPP2R5E*)	0.4064
	Pragmatic language impairment	15		17 (13)	−0.0129 (cg16518861 located in *NFKBIA*)	−0.0251 (cg06491415 located in *RPS6KA2*)	−0.0179	0.1007 (cg08223235 located in *BCL2*)	0.2519 (cg05651511 located in *RPTOR*)	0.1531
		16		11 (8)	−0.0203 (cg07499142 in *PIK3CD*)	−0.0506 (cg01320698 in *PIK3CD*)	−0.0287	0.1544 (cg18758433 in *RPTOR*)	0.3609 (cg01320698 in *PIK3CD*)	0.2339
		19		11 (10)	−0.0500 (cg18758433 in *RPTOR*)	−0.0929 (cg26360197 in *RPTOR*)	−0.0777	0.1834 (cg18758433 in *RPTOR*)	0.4273 (cg23575275 in *CDKN1A*)	0.3277
	Rigid personality	15								
		16		1 (1)	−0.0283 (cg26601310 located in PRR5L)			0.2867 (cg26601310 in PRR5L)		
		19								
	Total BAP score	15		18 (14)	−0.0138 (cg07499142 located in *PIK3CD*)	−0.0237 (cg06491415 located in *RPS6KA2*)	−0.0189	0.1512 (cg07499142 located in *PIK3CD*)	0.3432 (cg05651511 located in *RPTOR*)	0.2256
		16		15 (11)	−0.0196 (cg17306848 in *PRKCH*)	−0.0381 (cg01320698 in *PIK3CD*)	−0.0258	0.1777 (cg13989999 in *BCL2L1*)	0.3834 (cg00689225 in *NFKBIA*)	0.2492
		19		4 (4)	−0.0640 (cg23575275 in *CDKN1A*)	−0.0785 (cg26360197 in *RPTOR*)	−0.0686	0.0578 (cg23575275 in *DKN1A*)	0.3849 (cg11833768 in *PPP2R5E*)	0.2823
Cognitive Appraisal	Aloof	15	61 (41)	39 (33)	−0.0876 (cg14001486 in *PRKCH*)	−0.2037 (cg04610450 in *PIK3R2*)	−0.1458	0.1121 (cg22666015 in *INPP5D*)	0.3008 (cg12873919 in *BCL2L1*)	0.1891
		16		31 (27)	−0.1370 (cg13916261 in *FNBP1*)	−0.3626 (cg18758433 in *RPTOR*)	−0.2278	0.1422 (cg00278517 in *PS6KA2*)	0.3870 (cg18758433 in *RPTOR*)	0.2166
		19		4 (3)	−0.3259 (cg02481000 in *PRKCZ*)	−0.3947 (cg22666015 in *INPP5D*)	−0.3633	0.2544 (cg00300046 in *PRKCZ*)	0.4497 (cg23575275 in *CDKN1A*)	0.3273
	Pragmatic language impairment	15		29 (24)	−0.0673 (cg25342409 in *PIK3R5*)	−0.1180 (cg08557970 in *RPS6KA2*)	−0.0897	0.0817 (cg25342409 in *PIK3R5*)	0.1867 (cg16994041 in *PRKAG2*)	0.1350
				*1 Positive* *Mediation*	*0.0531* *cg08257293 in BCL2L1*			*0.0966* *cg08257293 in BCL2L1*		
		16		14 (13)	−0.0940 (cg00319686 in *MAP2K1*)	−0.1519 (cg17904575 in *PPP2R5C*)	−0.1259	0.1533 (cg26601310 in *PRR5L*)	0.2632 (cg02015053 in *EIF3J*)	0.1975
		19		22 (18)	−0.1339 (cg24585377 in *RPS6KA1*)	−0.3377 (cg02481000 in *PRKCZ*)	−0.2165	0.2089 (cg24585377 in *PS6KA1*)	0.7253 (cg02481000 in *PRKCZ*)	0.3304
	Rigid personality	15								
		16		1 (1)	−0.1577 cg13916261 in FNBP1			0.2440 cg13916261 in FNBP1		
		19								
	Total BAP score	15		31 (28)	−0.1242 (cg04610450 in *PIK3R2*)	−0.0570 (cg23174662 in *HIF1A*)	−0.0925	0.0889 (cg25342409 in *PIK3R5*)	0.2234 (cg12873919 in *BCL2L1*)	0.1454
		16		10 (9)	−0.1123 (cg00319686 in *MAP2K1*)	−0.1897 (cg18758433 in *RPTOR*)	−0.1468	0.1772 (cg05581469 in *RKAG1*)	0.2390 (cg18758433 in *RPTOR*)	0.2071
		19		4 (3)	−0.1966 (cg23575275 in *CDKN1A*)	−0.3006 (cg22666015 in *INPP5D*)	−0.2571	0.2741 (cg23575275 in *DKN1A*)	0.4031 (cg02481000 in *PRKCZ*)	0.3406

## Data Availability

The data presented in this study are available on request from the corresponding author due to research ethics board restrictions.

## Supplementary Materials

The following supporting information can be downloaded at https://www.mdpi.com/article/10.3390/ijms26199468/s1.

## Abbreviations

AKT	AKT Serine/Threonine Kinase
ASD	Autism Spectrum Disorder
BCL2L1	BCL2 Like
DNA	Deoxyribonucleic Acid
FNBP1	Formin Binding Protein 1
IES-R	Impact of Event Scale—Revised
mTOR	mechanistic target of rapamycin
NFKBIA	NFKB Inhibitor Alpha
P13K	phosphatidylinositol 3-kinase
PBMCs	Peripheral Blood Mononuclear Cells
PIK3CD	Phosphatidylinositol-4,5-Bisphosphate 3-Kinase Catalytic Subunit Delta
PNMS	Prenatal Maternal Stress
PPP2R5C	Protein Phosphatase 2 Regulatory Subunit B’Gamma
PPP2R5E	Protein Phosphatase 2 Regulatory Subunit B’Epsilon
PRKCH	Protein Kinase C Eta
PRRSL	Proline Rich 5 Like
PTSD	Post-Traumatic Stress Disorder
RPTOR	Regulatory Associated Protein of mTOR Complex 1
